# Lessons from the history of *Agave*: ecological and cultural context for valuation of CAM

**DOI:** 10.1093/aob/mcad072

**Published:** 2023-06-06

**Authors:** Sarah C Davis, Hector G Ortiz-Cano

**Affiliations:** Voinovich School of Leadership and Public Service, Ohio University, Building 22 The Ridges, Athens, OH 45701, USA; Holden Arboretum, 9550 Sperry Road, Kirkland, OH 44094, USA

**Keywords:** *Agave angustifolia*, *Agave tequilana*, *Agave americana*, tequila, bacanora, mescal, pulque, traditional knowledge, pre-Columbian, climate change, resilient agriculture, crassulacean acid metabolism

## Abstract

**Background and Scope:**

Crassulacean acid metabolism (CAM) is an intriguing physiological adaptation in plants that are widespread throughout many ecosystems. Despite the relatively recent mechanistic understanding of CAM in plant physiology, evidence from historical records suggests that ancient cultures in the Americas also recognized the value of CAM plants. *Agave* species, in particular, have a rich cultural legacy that provides a foundation for commercially valued products. Here, we review that legacy and potential relationships between ancient values and the needs of modern-day climate adaptation strategies.

**Conclusions:**

There are many products that can be produced from *Agave* species, including food, sugar, fibre and medicines. Traditional knowledge about agricultural management and preparation of plant products can be combined with new ecophysiological knowledge and agronomic techniques to develop these resources in the borderland region of the southwestern USA and Mexico. Historical records of pre-Columbian practices in the Sonoran desert and remnants of centuries-old agriculture in Baja California and Sonora demonstrate the climate resilience of *Agave* agriculture. Commercial growth of both tequila and bacanora indicates the potential for large-scale production today, but also underscores the importance of adopting regenerative agricultural practices to accomplish environmentally sustainable production. Recent international recognition of the Appellation of Origin for several *Agave* species produced for spirits in Mexico might provide opportunities for agricultural diversification. In contrast, fibre is currently produced from several *Agave* species on many continents. Projections of growth with future climate change suggest that *Agave* spp. will be viable alternatives for commodity crops that suffer declines during drought and increased temperatures. Historical cultivation of *Agave* affirms that these CAM plants can supply sugar, soft and hard fibres, medicines and food supplements.

## INTRODUCTION

Crassulacean acid metabolism (CAM) is one of the most fascinating physiological mechanisms observed in plants. More than 34 families include species that use CAM, representing 6 % of all vascular plants (Winter and Smith, 1996). CAM plants have a 24 h cycling of carbon that provides the chemical energy supply for photosynthetic light reactions even during times when stomata are closed. This has tremendous advantages for plants growing in hot and dry climates, but it is also beneficial for some aquatic plants, and CAM plants inhabit regions with a wide range of climatic conditions (Gilman and Edwards, 2020). The advantages of CAM have earned some CAM plants cultural significance throughout history by offering provisions in extreme climatic conditions.

Reflecting on the history of CAM research, which has provided profound insight into the plasticity of photosynthetic mechanisms, it is also appropriate to consider the many cultural associations with CAM plants. Although CAM plants are underrepresented in commercial agricultural production, several species make significant contributions to agricultural resources (Davis *et al.*, 2019). For example, pineapples [*Ananas comosus* (L.) Merrill] are globally traded fruits highly valued in food systems (Bartholomew *et al.*, 2002; Ming *et al.*, 2015), aloe varieties (360 *Aloe* spp.) are globally renowned for their medicinal qualities (Grindlay and Reynolds, 1986; Katerere, 2018), vanilla (107 *Vanilla* spp.) is a prized culinary spice (Fouché and Jouve, 1999; Cameron, 2011), blue agave (*Agave tequilana* Weber var. azul) is well known for the spirit tequila (Valenzuela-Zapata, 1997), and many types of orchids (family Orchidaceae with >29 000 spp.) are revered in ornamental horticulture (Arditti, 1992; Silvera *et al.*, 2009; De *et al.*, 2014). All these highly valued plants use CAM, although notably, the many varieties of orchids range in CAM expression from no CAM to strong CAM (Silvera *et al.*, 2009, 2010). These agriculturally important CAM plants have historical legacies associated with cultural identities in different regions of the world (e.g. Valenzuela-Zapata and Nabhan, 2004; Cameron, 2011).

All species in the *Agave* genus, >200 in total, are presumed to be obligate CAM plants, and this taxonomic group has a prominent cultural identity; the goddess Mayahuel still permeates art, symbols and even advertising as the divine figure embodied in *Agave* plants (Radding, 2012). Although the early works by Klaus Winter and others (e.g. Park Nobel especially for *Agave*) highlighted the significance of CAM in plant physiology, the cultural significance of this specially adapted group of the plants was evident in history well before western science documented it. Large-scale production of *Agave* species for human consumption is evident in North America even during pre-Columbian times (e.g. Ortiz-Cano *et al.*, 2020). Researchers such as Gentry (1982, 2004), Valenzuela-Zapata and Nabhan (2004), Vargas-Ponce *et al.* (2007, 2009) and others have documented the many landraces of *Agave* and their relationship to cultural history in the Americas. A text edited by Colunga-GarcíaMarín *et al.* (2007) provides a thorough account of traditional uses of *Agave*, which range from food and medicine to fibre and construction materials. The rich history of this genera thus lends itself well to an emerging model for rediscovery of natural resources through traditional ecological knowledge (Kimmerer, 2012). Rediscovery of traditional ecological knowledge has the power to guide diversification strategies for sustainable agriculture with changing climatic conditions, sometimes with equal or greater impact than biotechnology (IPES, 2016).

This paper reviews several *Agave* species, their traditional uses, cultural values and the potential for application to future agricultural production. Although we learned much about the amazing potential of CAM photosynthesis over the last 50 years that is being used for biotechnological advances in photosynthetic systems (e.g. Yuan *et al.*, 2020), there is also tremendous value associated with the careful cataloguing of plants that exhibit CAM traits in the wild (e.g. Winter, 2019). With the insight from new mechanistic knowledge about CAM, additional light can be shed on the cultural value of CAM plants, both historically and for the future. The history of *Agave* use might have something to teach us as we reach critical thresholds for climate change.

## OVERVIEW OF HISTORICAL USES AND CULTIVATION OF *AGAVE*

**Fig. 1.**
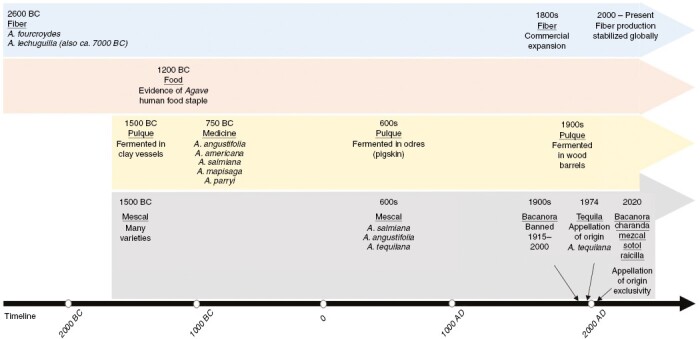
Time lines of documented uses of *Agave* species for food (pink); fibre (blue); pulque and medicines (yellow); and mescals and other spirits with an Appellation of Origin in Mexico (grey).

Most *Agave* species are native to Mexico, with 75 % of the 200 known species present throughout the country (García-Mendoza, 2002; Eguiarte *et al.*, 2021). The uses of *Agave* include food, fibre, construction materials, fermented beverages, antimicrobial agents, anti-inflammatory agents, dietary supplements and biofuel (e.g. Gentry, 1982; Colunga-GarcíaMarín *et al.*, 2007; Davis *et al.*, 2019). In the native range of *Agave* in Mexico, some communities harvest plants from the wild (e.g. Martínez-Palacios *et al.*, 1999) and others rely on traditional semi-domestication strategies, with subsistence farming of *Agave* plants in both forest and grassland areas. The density of plants in semi-domestication ranges from 20 to 3000 plants per hectare (Martínez-Palacios *et al.*, 1999). Historically, these plants were used to make everything from brooms to sandals to beverages, but the most common traditional use that persists today is for the fermented beverage pulque (Escalante *et al.*, 2016). Pulque has a lower alcohol content than the internationally recognized liquors (mescals) and purported health benefits in some cases (Escalante *et al.*, 2016). Industrial production of mescal (as reviewed in a section below) followed a long tradition of fermenting wild and domesticated varieties of *Agave* plants (Fig. 1).

Fibre production from *Agave fourcroydes* also has a long history in Mexico. Henequen, sometimes also called sisal, was produced during the Mayan era (Colunga-GarcíaMarín and May-Pat, 1993, 1997; Colunga-GarcíaMarín, 2007). This fibre provided the foundation for a rope and twine industry that expanded in the 1800s and peaked in the mid-1900s (Evans, 2007). After synthetic fibre production began, the *Agave* fibre industry declined substantially (Evans, 2007; Davis and Long, 2014)*. Agave lechuguilla* also provides fibre materials in Mexico, but is primarily marketed in local rural economies, especially in the region of Coahuila where it borders Tamaulipas and Texas (Castillo-Quiroz *et al.*, 2012). *Agave sisalana* has been produced commercially for fibre, originating in Mexico and then developed agriculturally throughout eastern Africa, India and Brazil (Gentry, 1982). Tanzania was the leading producer of sisal in the 1960s, but this industry was displaced by synthetic fibre production (Davis and Long, 2014; Davis, 2022). Today, Brazil has the greatest agricultural production of *Agave* fibre (Davis, 2022).

There is evidence of *Agave* use in North America during prehistoric times. Hammerl *et al.* (2015) reported *Agave* dental quids found in human remains, dated between 1200 and 1400 years BC, at the Loma San Gabriel in Durango, Mexico (Fig. 1). In Mesoamerica and Aridamerica, *Agave* cultivation was a subsistence strategy amongst pre-Columbian groups (Colunga-GarcíaMarín, 1997; Nabhan, 1985; Nabhan *et al.*, 2020). During pre-Hispanic times, *Agave* was cultivated in a similar manner, with annual seed crops, in Mesoamerica (Anderies *et al.*, 2008; Nabhan *et al.*, 2020). The cultivation and economic importance of ancient *Agave* or ‘Maguey’ in Mesoamerica can be traced to the Aztec empire in the highlands of central Mexico, where significant improvement in cultivation practices occurred at ~759 AD, and further south in the Mayan region at ~700 and 600 AD (Nichols *et al.*, 2000). In the Aridamerica region in south-central Arizona, cultivation of *Agave* as a staple crop by the Hohokam occurred at ~1150 AD (Fish and Fish, 1992). Likewise, Trombold (2017) reported *Agave* cultivation in north-central Mexico, in the Zacatecas region at La Quemada archaeological site, between ~1000 and ~1200 AD.

Evidence from pre-Hispanic times suggests that *Agave* cultivation intensified as a strategy to ensure food availability during times of drought and famine in both Mesoamerica and arid regions of the Sonoran and Chihuahuan deserts (Nichols *et al.*, 2000; Anderies *et al.*, 2008; Trombold, 2017). Pre-Hispanic archaeological sites of *Agave* are often accompanied by evidence of roasting pits or earthen ovens for *Agave* and food preparation. Remnants of *Agave* industries for goods (i.e. fibre, clothes) have been found at sites in western Mexico Colima, southern Zacatecas, Paquime and south-central Arizona regions (Zizumbo-Villarreal *et al.*, 2013; Fish and Fish, 2014; Minnis and Whalen, 2015). Today, as in pre-Hispanic times, several products made from *Agave* continue as part of Indigenous traditions in rural areas from southern to northern Mexico and borderlands with the USA (Esparza-Ibarra *et al.*, 2015; Ortiz-Cano *et al.*, 2020). Colunga-GarcíaMarín and May-Pat (1993) highlighted the different parts of *Agave* and subproducts in eight categories of traditional uses: drinks, clothes, construction, food, ornamental, domestic uses, agriculture and livestock.

The physiological advantages of CAM are key factors defining the natural and cultural history of *Agave* in different regions across Mexico and in the borderlands of Mexico and the USA (Gentry, 1972, 1982; Yetman *et al.*, 2002; Ortiz-Cano *et al.*, 2020; Eguiarte *et al.*, 2021). Remains of *Agave* goods at archaeological sites provide evidence of the importance of the natural diversity of the genus and its uses in pre-Hispanic times (Fish and Fish, 1992). The water-use efficiency, soluble carbohydrates, waxy cuticles and strong fibres of *Agave* are all traits associated with CAM; these products from *Agave* became embedded in the traditional practices amongst tribal groups and continue in modern times in rural Mexico and borderland regions (Bruman, 2000; Yetman *et al.*, 2002; Cervantes Mendívil *et al.*, 2007; Fish and Fish, 2014). Today, the most well-known examples of commercial and traditional use of *Agave* are for spirit drinks and fibre (Fig. 1).

## DETAILED HISTORY OF *AGAVE* CULTIVATION FOR FIBRE

The strong *Agave* fibres, grown by these CAM plants to support the weight of large succulent leaves, have been important for Indigenous people of North America since before recorded history. Agave fibre can be traced in history from ancestral people to modern Indigenous groups and geographically from southern Mexico to northern Mexico and borderlands with the USA. Andres Perez de Ribas, a Jesuit monk, reported that Indigenous people used *Agave* fibre for clothes between 1591 and 1620 in northern Sinaloa region (Rojo and Radding, 2019). The Chichimecas, who lived in several regions of northern Mexico for ~9000 years with few lifestyle changes until European arrival in America, used *Agave* extensively for fibre (Ruíz and Orozco, 2023). Fibre from *A. lechuguilla* was a valuable resource for the Chichimecas and was used to build traps, fishing nets, ropes, sandals, clothing, bow strings and string for tying arrowheads.

Currently in north-west Mexico, fibre extraction from *Agave* among Indigenous people occurs only rarely. Engerrand (1936) reported that the Tarahumara tribe in Chihuahua Mexico substituted the use of *Agave* fibre for cotton or wool. In southern Sonora, a few people in the Mayo tribe continue to extract fibre from *Agave* to produce rope and burlap bags (Yetman *et al.*, 2002). In rural communities of the Chihuahuan Desert, particularly in the state of Coahuila, fibre extraction from *A. lechuguilla* produces important revenues for local economies in the region (Castillo-Quiroz *et al.*, 2014). A census from 2005 estimated that 52 000 families produced fibre from *A. lechuguilla* as an economic activity in the Chihuahuan desert (López-Serrano *et al.*, 2021). Small groups of Indigenous people, such as the Otomi people in Ixmiquilpan Hidalgo, have preserved the tradition of extracting fibre from *A. lechuguilla* since pre-Hispanic times (CCMSS, 2022).

Yucatan is the centre of biodiversity of *A. fourcroydes*, which was used to produce henequen fibre by the Mayans (Garcia-ColungaMarin and May-Pat, 1993). In the Mayan region of southern Mexico, it has been hypothesized that Mayans used *Agave* ~2600 BC (Turner and Miksicek, 1984). Historically, the major fibre industry from *Agave* was the production of henequen at large colonial haciendas and plantations in southern Mexico (Wells, 1982). The Spanish villages in Yucatan date back to colonial times in the 1500s (Barteet, 2015). Spaniards in the Yucatan region occupied the Mayan land and developed irrigation technology for agriculture, such as the invention of the noria system (wells operated by working animals, typically mules or donkeys) (Barteet, 2015). The Mexican industry of fibre production intensified and reached its maximum between 1870 and 1901 owing to the mechanization of fibre extraction in the late 1800s and early 1900s (Garcia-ColungaMarin and May-Pat, 1993).

Since colonial times, the henequen industry relied on Mayan peons (Indigenous people enslaved and tied to an owner). Even after the Mexican revolution and redistribution of land, the henequen industry was controlled by landholders (Wells, 1982) who exploited thousands of Indigenous people in Mexico (Arias López *et al.*, 2013). Espinoza (2007) suggested that the success of the fibre industry in southern Mexico was attributable to the historical domination of European and upper-class social groups who enslaved Indigenous societies in Mexico (Lundberg, 2019). It has been reported that 300–400 hacendados (hacienda owners) owned >1000 henequen haciendas (Wells, 1982). A few families controlled and monopolized the henequen industry in Yucatan, such as the Peón family, who owned land titles ≤7000 ha in size since 1841 (Wells, 1982). In 1914, the largest plantation of henequen produced only 196 tons of fibre (Wells, 1982). By 2017, the production of fibre reported was 12 813 tons annually across 6461 ha cultivated with *A. fourcroydes*, generating a revenue of 59.2 million pesos in Yucatan (Wells, 1982; SEGOB, 2022*b*).

In the first half of the 1800s, conflict between the government and the Yaqui tribe in southern Sonora resulted in entire families of Yaquis being expelled and deprived of their land, forcing them to move and work in the Yucatan region (Arias López *et al.*, 2013). Yaqui families were captured, transported in trains and integrated into henequen plantations at haciendas in the Yucatan region. In the 1900s, it has been estimated that 8000 Yaquis were relocated to the Yucatan peninsula to work at henequen and sugarcane plantations or salt ranches (Arias López *et al.*, 2013). The Yaqui history in Yucatan has been traced and recorded by Teodoro Buitimea Flores, a member of the Yaqui tribe in charge of recovering the history of the Yaqui in Mexico. Prior historical accounts have overlooked the suffering of Indigenous children, both male and female, in Yucatan and other industries of *Agave* (T. Buitimea Flores, Superior Technological Institute of Cajeme (ITESCA), Outreach Extension Office Vicam, Son, MX, pers. comm.).

The terms ‘henequen’ and ‘sisal’ have been exchangeable to name hard fibre of *A. fourcroydes* and *A. sisalana*. In the late 1800s, *A. sisalana* fibre attracted the British and Germans, and samples from Mexico were sent to botanical gardens in Europe, then later propagated and introduced in the German colonies for *Agave* fibre in Eastern Africa (Brockway, 1979). Brown (2002) indicated that 1000 bulbils of *A. sisalana* were sent to Florida in 1893 and from there to Germany. Later, *Agave* was introduced to the Phillipines, Brazil, India and other countries in Asia (Brown, 2002). The distribution of *A. sisalana* in southern Mexico is not clear, but it has been hypothesized that it was propagated and used by Indigenous people in villages around the Chiapas region (Gentry, 1982, 2004; Brown, 2002). In 2021, the sisal industry produced 300 000 tons of fibre, valued at 75 million USD, and the major producers are reportedly Brazil (120 000 tons), Tanzania (30 000 tons) and Kenya (25 000 tons) (DNFI, 2020). Although the global fibre industry peaked in the 1960s, then declined as synthetic fibres were introduced (Davis and Long, 2014), *Agave* fibre production has remained relatively consistent in Mexico over the last decade, nationally ranking fourth in global production (Davis, 2022).

## REGIONALIZED PRODUCTS FROM *AGAVE* SPECIES IN THEIR HISTORICAL RANGES

Amongst products made of *Agave*, spirit drinks have the most defined commercial and geographical history in Mexico and the southwestern US borderland regions (Salazar Solano, 2007; Salazar Solano and Mungaray Lagarda, 2009; Pérez-Akaki *et al.*, 2021). The process of CAM in *Agave* plants leads to a reliable accumulation of soluble carbohydrates in the stem and leaf tissues (e.g. Jones *et al.*, 2020), and these sugars are easily fermented to yield alcohol. Throughout history, farmers have developed their signature drinks using different endemic species available in the arid, semi-arid and temperate regions (Bruman, 2000). The most distinctive examples of regionalized *Agave* spirits in their historical commercial ranges are pulque, mescal and tequila (Bruman, 2000; Serra and Carlos, 2010; Escalante *et al.*, 2016). Beginning in pre-Hispanic times, pulque was made of *Agave salmiana*, *Agave atrovirens* and *Agave mapisaga* by the Tlaxcaltecas, Otomi, Toltecs and Aztecs (Escalante *et al.*, 2016). Pulque is likely to be the oldest drink consumed from *Agave* in America, and its crafting has been estimated to originate 3500 years ago (Escalante *et al.*, 2016).

**Fig. 2.**
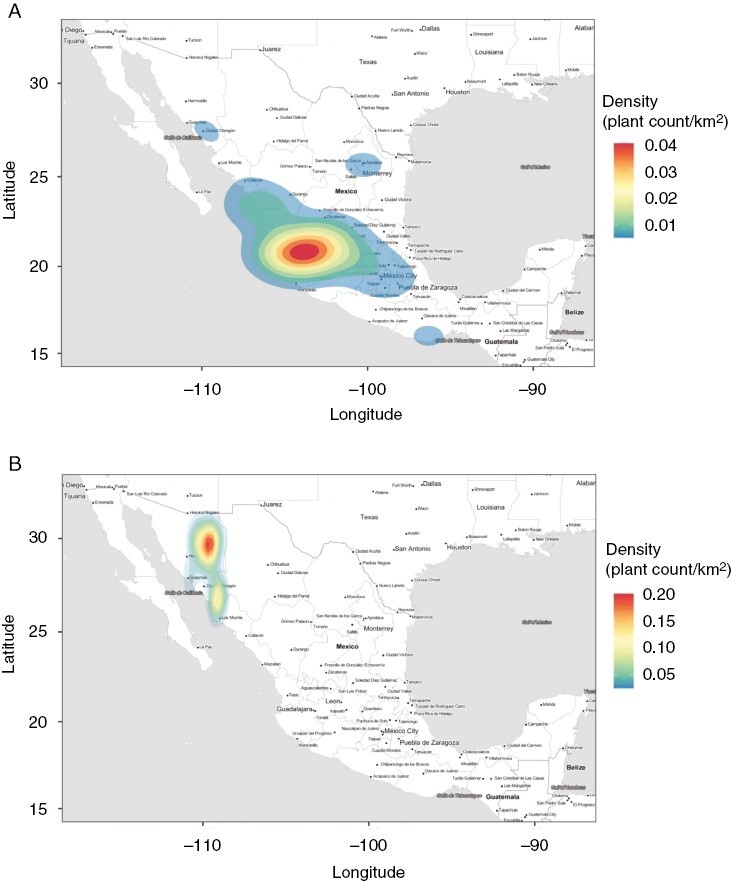
Regional Appellation of Origins denoted with a heat map of density of occurrences (plant counts per kilometre squared) indicating centres of cultivation (in red) for *Agave tequilana* used for tequila (A) and *Agave angustifolia* used for bacanora (B) throughout Mexico. (Data source: GBIF, 2022; map generated with GADM CC-BY, https://gadm.org/license.html.)

In recent decades, the Appellation of Origin, also called Designation of Origin, for *Agave* spirits has helped distilled *Agave* drinks to reach their maximum commercial and cultural–legal recognition (Pérez-Akaki *et al.*, 2021). In Mexico, the Designation of Origin system for *Agave* spirits is similar to those used in Europe in the wine and food industry that protect regional products from specific geographical ranges, with particular artisanal processes, varieties and history, providing economic alternatives amongst regional farmers (Ortiz-Cano *et al.*, 2020). In western Mexico, tequila was the first *Agave* spirit recognized through a Designation of Origin and, internationally, an Appellation of Origin (Fig. 1; Bowen, 2015; Pérez-Akaki *et al.*, 2021). Another example is the mescal named bacanora, produced from *Agave angustifolia* in Sonora, Mexico, which in recent years has gained international presence in Europe amongst the highest quality international spirits (Arizona Public Media, 2020). As the signature for bacanora, the Designation of Origin establishes the exclusive use of *A. angustifolia* plants for its production in 35 municipalities along the Sierra Madre Occidental mountain range in Sonora, Mexico (Fig. 2). Likewise, the Designation of Origin establishes that tequila can be produced legally only from *Agave tequilana* Weber var. azul in 125 municipalities in the State of Jalisco, 30 in Michoacan, 7 in Guanajuato, 8 in Nayarit and 11 in Tamaulipas (Fig. 2; Consejo Regulador del Tequila, 2022).

The Designation of Origins for *Agave* spirits is recognized internationally under the Lisbon Agreement for the Protection of Appellation of Origin, and the registered Appellation of Origin has attracted Mexican producers of *Agave* to international markets. The increasing international interest and demand for *Agave* spirits has also increased the revenues from the tequila and mescal industry in Mexico. In 2021, the tequila industry reached its maximum commercial production in history, producing 527 million L. Internationally, the USA had the highest demand, selling $5200 million USD worth of tequila. In 2020, 374 million L of tequila were produced from 20 000 ha cultivated with *A. tequilana* Weber var. azul, with yields of 77.6 tons per hectare (SEGOB, 2022*a*; Statista, 2022). Likewise, in 2021 the mescal industry reached a new historical maximum production of 8.1 million L.

Although bacanora was the earliest of the *Agave* spirits to be traded internationally, and there are many other spirits produced through traditional practices, the tequila industry is currently the largest and most well known. In the following sections, we review the colonial period that changed agricultural production of *Agave* in Mexico, provide a history of changes associated with the development of tequila, then provide a historical review of bacanora development for comparison.

### Agave *regional developments during colonial Spanish period*

When Spaniards arrived in Mesoamerica during the early 1500s, they found that Indigenous people produced goods and drinks made of *Agave* (Walton, 1977; Bruman, 2000; De León Meza, 2017). In central Mexico, the Spanish found that people living in cities also cultivated *Agave*, while nomadic tribes consumed *Agave* extensively as a staple food and used it to produce goods (e.g. cloth, rope, bags; Evans, 1992; Gutiérrez-Coronado *et al.*, 2007; Serra and Carlos, 2010). Nomadic Chichimecas and Tlaxcaltecas harvested and roasted wild *Agave* or maguey (mixcali, mescal) in small ovens (Sánchez Soto, 2016). The Aztecs cultivated *Agave* in terraces on piedmont slopes in the valley of Chihuactepan, a region that they had occupied since 1000 AD (Córdova and Parsons, 1997; Evans, 2007). During the second half of the 1500s and into the early 1600s, the Spanish explored western Mexico with the help of Indigenous allies (Tlaxcaltecas, Huejotzingas and Purepechas), and they observed that the Caxcanes and the Tecuexes consumed *Agave* although they did not cultivate it (De León Meza, 2017).

The Spaniards found water and large extensions of fertile land that was suitable for agriculture and livestock in regions of western and northern Mexico (Tercero, 1936). In western Mexico, they recorded the arid regions around the volcano in what is now Jalisco state, where *Agave* was grown extensively by the Indigenous people (Valenzuela-Zapata and Nabhan, 2004). The Spanish strategy of establishing Franciscan and Jesuit parishes at missions, cofradias and villages enabled agriculture in the western and northern regions (Chance and Taylor, 1985; De León Meza, 2017). Jalisco continues to be the primary growing region for *A. tequilana* today.

European and Indigenous agricultural practices enabled the introduction of seedlings, grains, trees, fruits, flowers, bulbs and root grafts along with techniques such as decks, terraces and gardens in the different regions of Mexico (Brown and Fournier, 2014). Colonial settlements enabled agriculture of Iberoamerican crops and intermixing of regional plants endemic to Mesoamerica and Aridamerica (Brown and Fournier, 2014; Saldaña and Colín, 2014). For example, during Spanish exploration campaigns to northern Mexico, aided by the Tlaxcaltecas, *Agave* species were introduced to arid regions of the Chihuahuan desert. The Tlaxcaltecas brought with them *A. salmiana*, which naturalized to San Luis Potosi, Saltillo and Zacatecas regions (Saldaña and Colín, 2014). Nowadays, *A. salmiana* populations are part of the landscapes of arid regions in northern Mexico (Gentry, 1982). The ecological signature of colonial Spanish crops and species intermixing in northern and western Mexico has been estimated to affect several thousand endemic species in the region (Saldaña and Colín, 2014).

Colonial trading routes established within the first 100 years of Spanish colonial occupation, such as the ‘El Camino Real’, which connected Aridamerica and central colonial regions, crossing regions of northern and western Mexico, helped Spaniards to identify endemic species, including *Agave* species, that were used later in the colonies (Saldaña and Colin, 2014). The ‘El Camino Real’ route favoured the mescal industry and Tequila region to cultivate *Agave* in monoculture (Saldaña and Colín, 2014). It has been reported that mescal wine was produced initially in western Mexico and that Jalisco later became the main region for mescal (Walton, 1977).

### Agave *cultivation in the Tequila region*

Jacques *et al.* (2003) hypothesized that the word Tequila relates to the two words ‘Titicuilas’ and ‘Tequitl’. The Titicuilas were a tribe that inhabited the piedmont of a volcano near the city of Tequila. The word Tequitl, from the Nahuatl language, means work. Historically since the 1800s, Tequila has become the prime region for cultivating *Agave* in the Americas. The Tequila region includes the states of Jalisco, Guanajuato, Nayarit, Michoacan and Tamaulipas (DOF, 2022*a*). In 1974, the Designation of Origin of Tequila was created to protect the spirits made of *A. tequilana* Weber var. azul in the Tequila region (DOF, 2022*a*).

Tequila has been broadly associated with Mexican National Identity (Bowen and Gaytan, 2012 ). In 2006, UNESCO declared human patrimony of the *Agave* landscapes in Tequila, at Amatitan, Arenal and Magdalena (UNESCO, 2022). Despite Spanish colonial policies that prohibited liquor production from *Agave* in the early New Spain, tequila has been produced for ~235 years and is one of the oldest industrial *Agave* Indigenous drinks crafted and distilled in North America (Jacques *et al.*, 2003). Tequila made of *A. tequilana* Weber var. azul originated from the integration of the Indigenous traditional ecological knowledge and European agricultural practices (Colunga-GarcíaMarín *et al.*, 2007; Valenzuela, 2011). Thus, modern-day tequila can be considered a mestizo drink (mestizo is commonly used in Latin America to denominate the cultural intermix between Indigenous and European people) (Walton, 1977). The accumulated knowledge since pre-Hispanic times for harvesting and selecting *Agave* traits, combined with European monoculture systems brought by the Spanish to America, enabled cultivation of *Agave* in the region of Tequila (Walton, 1977; Cedeño Cruz, 2003).

Evidence of cultivation of *Agave* in Tequila goes back to 1726, with the story of Luis Clemente Gonzalez, an Indigenous tribal leader who inherited 500 *Agave* plants in the Amatitan region (Tena Meza *et al.*, 2015). Spanish interest in cultivating *Agave* started during the period of colonial intendancies, when the Spanish monarchy intensified control over the peninsular Spaniards after the second half of the 1700s (De León Meza, 2017). The rights for mescal and cultivation of *Agave* were provided to Prudencio Cuervo, who owned the Haciendas de San Martin and Guadalupe. At the hacienda San Martin in 1756, the first alambique (copper distillatory) was reported for distilling mescal (De León Meza, 2017). The hacienda San Nicolas, owned by Rafael Montaño in 1777, recorded 2810 cultivated *Agave* plants and 500 plants that were mature and ready to be harvested (Goyas Mejía, 2012).

Although the main production in these haciendas was sugarcane, Rene De León Meza (2017) indicated that, in the Hacienda of San Martin inventory of 1787, there were 50 000 cultivated *Agave* plants, and by 1801 the number had increased to 364 407 plants. In the late 1800s, different haciendas produced thousands of litres of tequila. For example, by 1888, the hacienda Cuisillos near Guadalajara produced 1000 cargas de mescal (colonial Spanish units), equivalent to 188 630 L of tequila (Goyas Mejía, 2012). After 212 years of the *Agave* industry in the Tequila region, by 1999 the production reached 800 tons of plants for tequila. In 2021, 75 % of the national production of *Agave* across Mexico was produced in the Tequila region, and 1 777 000 tons of *A. tequilana* plants were produced (Mexicampo, 2022).

### Botanical history of Agave tequilana

Selection of *Agave* species occurred in pre-Hispanic times by Indigenous Mesoamericans and Aridamericans (Evans, 1992; Trombold, 2017). Pre-Columbian Indigenous groups knew the differences between species of *Agave* in the different regions of Mesoamerica and Aridamerica and could identify and select species based on traits (e.g. leaf colour, phenology) (Gentry, 1982). They domesticated species and naturalized species to different geographical ranges in Mesoamerica and Aridamerica (Gentry, 1982). Some species, such as *A. tequilana* Weber var. azul, evolved from *A. angustifolia* (Rodriguez Garay *et al.*, 2009).

The botanical history of the *Agave* genus started during early Spanish occupation of Mesoamerica and Europe in the mid-1500s. The first records of the ecology of *Agave* in colonial times can be found in the Codex Nutal, Florentino and Borgia (Jacques *et al.*, 2003). In Europe, Carolus Clusius, a French naturalist from the Leiden Botanical Garden, was attracted to *Agave* species introduced in Spain; *Agave* plants were among the first plants to be classified botanically, investigated and cultivated for research purposes (Ubrizsy in Savoia and Heniger, 1983). Carl Linnaeus published the first description of *Agave americana* in 1753 in the *Species Plantarum* (Trelease, 1908; Muriá, 2012). At the end of the 1700s, in Tequila, monocultures of *Agave* started with the beginning of commercial *Agave* production in North America. Don Cenobio Sauza introduced tequila to the USA for the first time at the Chicago World’s Fair in 1893 (Academia Patrón, 2022). In the early 1900s, the French naturalist Leon Diguet published the first book describing the cultivation procedures for *A. tequilana* in the Tequila region (Diguet, 1910). Today, several Mexican states and regions, with a variety of climatic and environmental conditions, have technical guides for cultivating *Agave* for tequila.

*Agave tequilana* Weber var. azul is one of the most studied *Agave* species in the world as a crop, with comprehensive evaluations taxonomically, physiologically, economically, ecologically and genetically. The high industrial demand for *A. tequilana* has contributed significantly to the motivation for exploring alternative propagation techniques. Since the early 2000s, tissue culture has been used to produce *Agave* plants (Jacques *et al.*, 2003). Recently, micropropagation and genetic transformation have been considered to be techniques with great potential to mass produce plants (Bautista-Montes *et al.*, 2022).

The French botanist Fréderik Albert Constantin Weber described *A. tequilana* F.A.C. Weber taxonomically in 1902 (Weber, 1902; GBIF, 2022). At that time, there were four varieties deemed superior for mescal use: chato, siquin, azul and pata de mula (Diguet, 1902). The blue cultivar (azul) of this species became the preferred crop plant for tequila (Gentry, 1982) and has been named *A. tequilana* Weber var. azul, as recognized by the Tequila Regulatory Council (Consejo Regulador del Tequila, 2022). Recently, *A. tequilana* has been reclassified as part of the Asparagaceae and Agavoideae family, which was formerly named Agavaceae (Jiménez-Barron *et al.*, 2020).

Another milestone in the history of *Agave* as a crop was reached by Nobel and Valenzuela (1987), who reported the environmental productivity index of *A. tequilana* in Jalisco. The environmental productivity index quantifies the relationship between growth and the light, temperature and water conditions of a site, predicting the effect of soil and environmental factors on CO_2_ uptake, and can be used to predict suitable regions for *Agave* cultivation (Garcia-Moya *et al.*, 2011). This model has been a keystone for understanding *Agave* species as an alternative drought-tolerant crop for current and future climate change (Davis *et al.*, 2011, 2017, 2021; Cushman *et al.*, 2015; Stewart, 2015; Owen *et al*, 2016; Ortiz-Cano *et al.*, 2020).

### Ecological challenges of tequila production

After >200 years of the tequila industry using *A. tequilana*, several ecological challenges have intensified in recent years. Gentry (1982) reported *A. tequilana* populations thriving in the wild in western Jalisco. In contrast, monocultures of *Agave* have disrupted pollinator corridors, particularly for nectarivorous bats, promoting international concern and a generalized problem that affects several *Agave* species in neighbouring regions of Tequila and in geographical regions across Mexico and the borderlands with the USA (Trejo-Salazar *et al.*, 2016; Burke *et al.*, 2021; IUCN, 2022). In addition, the low genetic diversity of *A. tequilana* monocultures threatens the genetic diversity of the species, particularly with current and expected climate change (Ruiz Mondragon *et al.*, 2022). The high industrial demand has caused conflict over water, compromising the capacity of *A. tequilana* as a crop in marginal and non-marginal lands (Fucikovsky, 2001). Moreover, industrial monopolies in the tequila industry have led to diseases, soil erosion, chemical pollution, shifts from traditional crops to *Agave*, and replacement of *Agave* landraces for mescal in the Tequila region, causing social injustice for small producers and Indigenous people (Zizumbo-Villarreal *et al.*, 2013; Bowen, 2015; Cabrera-Toledo *et al.*, 2022).

### Agave *cultivation in the Bacanora region*

Historically, domestication, cultivation and commercial uses of *Agave* have occurred in the biodiversity centres of *Agave* in northern Mexico and the borderlands with the USA (Eguiarte *et al.*, 2021). Hodgson *et al.* (2018) and Hodgson and Salywon (2013) found several *Agave* species domesticated by pre-Columbian people in central and southern Arizona. Gentry (1972) described the wide geographical range of *A. angustifolia* across Sonora, Mexico and commented about the long cultural history amongst Indigenous groups who make bacanora in the Sierra Madre Occidental in Sonora, Mexico. Salazar Solano (2007) suggested that the mescal bacanora received its name from Bacanora, Sonora, a town in the Sierra Madre Occidental, and it originated as part of the Opata traditions called Vitzo or Cuviso (Gutiérrez-Coronado *et al.*, 2007).

Historically, bacanora originated in the traditional knowledge and ancestral techniques practised by the Opata in Sonora (Shaul and Hill, 1998; Cervantes Mendívil *et al.*, 2007). The Opata were culturally connected with other populations in the Sonoran Desert, such as the Tepiman, the Yuman and the Hohokam, who cultivated *Agave* in central-southern Arizona (Shaul and Hill, 1998). The artisanal crafting methods for the baking, floral stalk emasculation and fermentation of *A. angustifolia* heads have been used since pre-Hispanic times (Fig. 1), and they remain current in the modern bacanora industry (Fish and Fish, 1992; Cervantes Mendívil *et al.*, 2007; Gutiérrez-Coronado et al., 2007). In the 17th century, Jesuit Spaniards documented consumption of fermented drinks from *Agave* amongst Piman people in the region (Gutiérrez-Coronado et al., 2007).

**Fig. 3.**
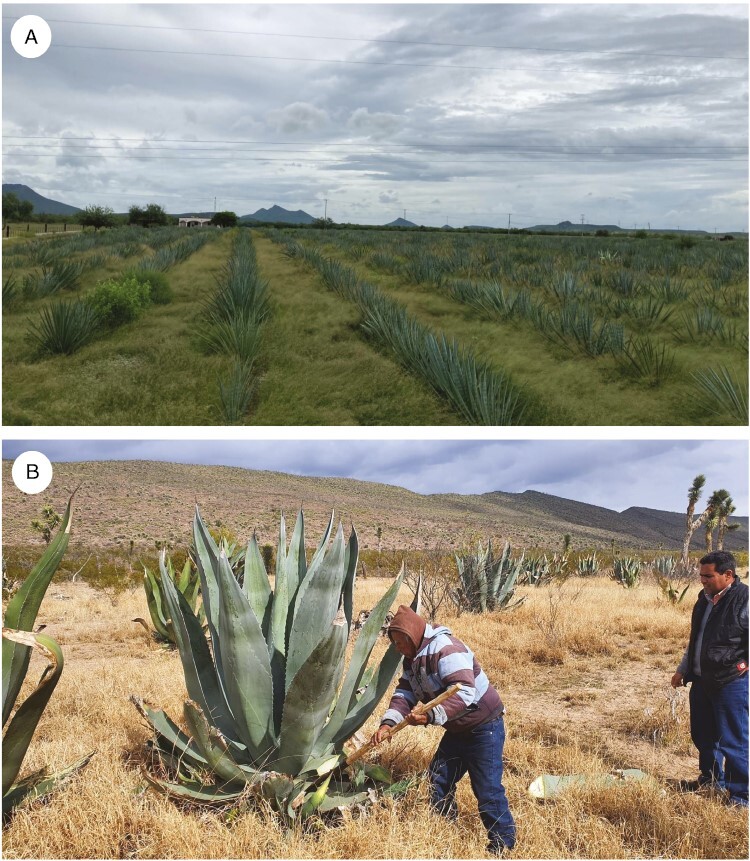
*Agave angustifolia* plantation (A) and *Agave salmania* plantation (B) in Sonora Mexico (Coahuilo). These plantations are well known for high-yielding plants that produce aguamiel (*Agave* sap). In B, Nazario Gonzalez (owner) and Professor Antonio Hernandez are pictured inspecting the plants. (Photographs courtesy of Dr Jose Antonio Hernandez Herrera, Universidad Antonio Narro.)

After the introduction of the alambique in Sonora, wild *A. angustifolia* became the feedstock of mescal in the region (Fig. 3). Production of Sonoran spirits, or aguardientes, began around the 1800s and included bacanora, lechuguilla and mezcal ceniza (Frisby Morales *et al.*, 2018). When La Reforma in the 1850s established rights and permits for artisanal mescal producers that freed them from taxation by the Catholic Church, a boost in the industry of bacanora followed. Between 1900 and 1910, the bacanora industry, with 75 distilleries, produced the majority of Sonoran mescals. In 10 years, production increased from 436 406 to 832 111 L of mescal, generating revenue of ~941 307 pesos. The current value of the volume of bacanora produced in the early 1900s is ~115 million pesos, ~$6 million USD.

The production of bacanora was prohibited from 1915 to 2000 (Salazar Solano, 2007). In 2000, the bacanora industry gained legal recognition through Designation of Origin (Fig. 2) and by the NOM-168-SCFI-2005 (DOF, 2022*b*). Today, with ~1000 producers, bacanora has reached its highest production in the last 20 years, with 300 000 L of mescal worth 70 million pesos or $3 636 570 USD (Consejo Regulador del Bacanora, 2015).

#### Ecological challenges of the bacanora industry.

The bacanora industry started in the early 1900s, and production depended on a combined system of producing plants in commercial plantations and harvesting wild *Agave* populations (Cervantes Mendívil *et al.*, 2007). Producers were aware that wild *Agave* could not provide enough material to sustain production (Salazar Solano, 2007). During 75 years of prohibition, illegal production of bacanora significantly reduced the regenerative capacity of wild *Agave* in Sonora (Cervantes Mendívil *et al.*, 2007). After the end of prohibition, the original system from the early 1900s for *A. angustifolia* production was reactivated in the bacanora industry. However, ecological sustainability and low wild *Agave* regeneration time remain major challenges to growth of the bacanora industry in Sonora (Núñez Noriega and Salazar Solano, 2009). Current climate science education and outreach programmes are needed in order to continue the *Agave* industry. Moreover, promotion of a more diversified spirit industry using different *Agave* species might reduce the ecological pressure on *A. angustifolia* in Sonora.

#### Bacanora industry in its historical range at the borderlands of Sonora and Arizona.

Mescal bacanora plays an important geographical role in the historical economy of the borderlands in Sonora, Mexico and Arizona, USA (Salazar Solano and Mungaray Lagarda, 2009). Arizona territory, annexed to the USA in 1863 during the early stages of the bacanora industry in Sonora (Argüello, 1988; Salazar Solano, 2007; Taylor, 2008; Massoth, 2016), resulted in the naturalization of a large population of Mexican citizens, with roots in Sonora, Mexico, to the Union of the USA (Taylor, 2008). Salazar Solano (2007) found 1891 records that reported trading and exporting of mescal along the borders of Sonora with Arizona. Bacanora was probably the first *Agave* spirit with international demand in the US territory. In the last 100 years, the flux of immigrants throughout the borderlands has increased the demand for mescal. In the borderlands with Arizona, the ‘nostalgic market’, driven by the preference of Sonoran immigrants for bacanora (Salazar Solano and Mungaray Lagarda, 2009), has increased the international demand for bacanora in the USA.

**Fig. 4.**
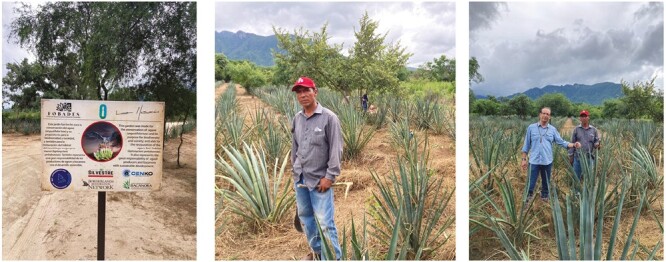
Commercial plantation for bacanora with agroforestry and conservation of *Agave angustifolia* populations supported by FOBADES and the Borderlands Restoration Network in Sonora, Mexico (sign pictured on the left). Dr Hector Ortiz-Cano (right) and Daniel Moroyoqui (centre and right) are pictured at the Javier Figueroa plantation in Alamos, Sonora, Mexico.

The history, value and ecology of bacanora in the borderlands have attracted the attention of Arizonans, particularly to research the potential for sustainable commercial production and conservation of *Agave* in the USA (McDaniel, 1985; Davis *et al.*, 2017). Current international cooperation across the borderlands includes conservation efforts by the University of Arizona Borderlands Restoration Network, the Fondo para el Bacanora para el Desarrollo Sostenible (FOBADES) and the Instituto Nacional de Investigaciones Agrícolas, Forestales y Pecuarias (INIFAP) in Sonora, Centro de Investigación en Alimentos y Desarrollo (CIAD). All these organizations have played a crucial role in studying ecological and sustainable strategies, with input from small farmers, which benefit the bacanora industry and wild *Agave* populations in Sonora and borderlands with Arizona (Fig. 4). Such cooperation is essential for a new mescal industry with less environmental impact. In the last 10 years, a new bacanora culture, with women leading the industry, has positioned bacanora as a competitor with the highest standard spirits in the European Union (Domínguez-Arista, 2020).

## REVIEW OF RECENT FIELD TRIALS OF *AGAVE* OUTSIDE THE HISTORICAL RANGE

Although there are many current examples of *Agave* species in agricultural production in Central and South America and in subtropical–tropical climates in Africa and Southeast Asia, there have also been recent studies to test the feasibility of *Agave* for agriculture outside the typical commercial range, in North America (Davis *et al.*, 2017) and Australia (Holtum *et al.*, 2011). A field experiment in Maricopa, Arizona (at latitude 33°N) indicated that *A. americana* was more tolerant of the occasional cold temperatures during winter months than both *A. tequilana* and *A. fourcroydes* (Davis *et al.*, 2017). The yields of *A. americana* were limited by a specialized pest, *Scyphophorus acupunctatus* (agave snout weevil), but reached 9.3 dry Mg ha^−1^ year^−1^, even with the pest, because the weevil attacks only plants that reach a certain size (Davis *et al.*, 2017). *Agave americana* also demonstrated tolerance of arid conditions at the site, persisting with as little as 300 mm of annual water input. In Australia, at a site near Ayr, Queensland (latitude 20°S), where annual precipitation is greater (947 mm) and winters are milder, *A. tequilana* Weber var. azul was very productive outside its home range (Yan *et al.*, 2020). Yields of 7.2–21.6 dry Mg ha^−1^ year^−1^ were reported at the site after 5 years, with nearly half of the biomass composed of sugar (Yan *et al.*, 2020).

Although the history of agricultural production in Mexico and South America reveals the importance of *Agave* species for human use, recent studies indicate that a much wider range is possible. In addition to the field trials described above in the USA and Australia, model projections indicate that *Agave* production could extend far beyond its current range (Owen *et al.*, 2016; Davis *et al.*, 2021). The growing range of *A. tequilana* is projected to expand with climate change, as warmer temperatures reduce the susceptibility to cold at higher latitudes (Owen *et al.*, 2016). The northern latitudinal range of *A. americana* is already greater than that of *A. tequilana*, but projections with climate change indicate that the range can extend even further north (Davis *et al.*, 2021).

Although the temperature tolerances for these species are remarkable owing to CAM (Nobel, 1996, Nobel *et al.*, 1998; Davis *et al.*, 2022), the drought tolerance of *Agave* species is perhaps even more important for climate adaptation in agricultural landscapes (Davis *et al.*, 2014, 2022). Where yields of current commodity crops are expected to decline (Bezner Kerr *et al.*, 2022), *Agave* species might thrive in the future. *Agave* has the potential to produce the same amount of fibre that cotton crops yield, with less than a tenth of the water requirement (Davis *et al.*, 2021). The theoretical yield of a CAM crop in drought conditions is 2.5 times greater than that of a C_4_ crop and 4.5 times greater than that of a C_3_ crop (Borland *et al.*, 2009; Davis *et al.*, 2014). The high concentration of soluble carbohydrates remains consistent in *Agave* biomass in conditions with and without drought (Jones *et al.*, 2020), suggesting that it can be a reliable source of calories, sweetener or bioethanol.

## PROJECTIONS FOR THE FUTURE, WITH CLIMATE CHANGE AND DIVERSIFICATION

The historic Paris Agreement achieved during the 21st Conference of Parties (COP21), organized as part of the United Nations Framework Convention on Climate Change, led to unprecedented commitments from the global community to address greenhouse gas emissions, but the COP27 in Egypt in November 2022 revealed that the world is not on target to limit warming in the atmosphere of the Earth. The reality, even with renewed and improved commitments, is that climate change will continue in the near future. Warming of ≥2 °C is likely, and it is essential to start evaluating the resilience of agricultural crops that provide essential resources. Productivity levels of many crops are decreasing because of climate change (Bezner Kerr *et al.*, 2022).

Recent model projections of potential production indicate that *A. americana* has the potential to expand in geographical range with climate change and could yield greater biomass, sugar and fibre even with +4 °C warming (Davis *et al.*, 2021). The CAM traits allow this species to thrive even with severe drought events and extreme temperatures. *Agave americana* tolerates temperatures ≤63 °C (Nobel and Smith, 1983) and, with as little as 300 mm annual precipitation, can achieve greater yields than a soybean field with prime soils and ample rainfall (Davis, 2022). CAM plants, such as *A. americana*, offer viability and resilience in a warmer world, thereby supporting climate adaptation.

Although they are not considered key commodity crops today, traditional uses of *Agave* might teach us something about their associated ecosystem services (Nabhan, 2013). In an analysis of mission records and agricultural biodiversity on the Baja Peninsula in Mexico, *Agave* species were discovered to have persisted over the last three centuries in small farming sites that span the latitudinal range of the peninsula (De Grenade and Nabhan, 2016). These sites have average annual precipitation ranging from 97 to 308 mm, with average annual maximum temperatures of ≤47 °C (De Grenade and Nabhan, 2016). *Agave* species were planted as one of the perennial food crops on small farms in these sites between 1697 and 1768, and they were found persisting in the same locations recently (De Grenade and Nabhan, 2016). This is supporting evidence of the potential for *Agave* species to serve climate adaptation goals for agriculture.

## TRADITIONAL KNOWLEDGE FOR FUTURE REGENERATIVE AGRICULTURE

Dryland farming systems enabled Pre-Columbian farmers to cultivate *Agave* in the driest regions of North America (Fish *et al.*, 1985; Mikulska, 2001; Garcia-Moya *et al.*, 2011; Fish and Fish, 2014; Trombold, 2017; Nabhan *et al.*, 2020). Water catchment techniques on slopes, rock mulching, rock alignments, terraces, nurse plants and intercropping are among the strategies used to cultivate *Agave* (Homburg and Sandor, 2011; Fish and Fish, 2014; Ortiz-Cano *et al.*, 2020). Records from the 16th and 17th centuries, in the Codex Florentino, illustrate cultivation of *Agave* by Indigenous farmers in late phases of Mesoamerica and the early Spanish colonial period in Mexico (Ortiz *et al.*, 2020). Colunga-GarcíaMarín *et al.* (2007) and Gentry (1982) suggested that the first cultivated commercial *Agave* plantations were established at the end of the 17th century. Modern *Agave* cultivation derives from ancestral agricultural practices (Ortiz-Cano *et al.*, 2020). Early pre-Columbian farmers observed the drought tolerance, monocarpic habit, biomass accumulation, sugar accumulation after floral stalk emasculation, sexual and asexual reproduction, and the productive potential of *Agave* species in the mosaic of climates across Mesoamerica and Aridamerica, which are now regions of Mexico and the southwestern USA (Adams and Adams, 1998; Cervantes Mendívil *et al.*, 2007; Nabhan *et al.*, 2020).

Ancestral dryland farmers understood the topography, soil fertility, rainfall water management, suitable crops and climate for dry regions (Fish and Fish, 1992, 2014; Ortiz-Cano *et al.*, 2020). Groups such as the Toltecas, Mexicas, Chalchihuites and the Hohokam made marginal lands productive by cultivating *Agave* (Mikulska, 2001; Fish and Fish, 2014; Trombold, 2017). Mikulska (2001) suggested that farmers from central Mexico in the Huaxteca region used microregions to adapt their agricultural strategies. Dry regions or marginal lands, with ≤500 mm precipitation, were used to cultivate corn and *Agave*, whereas areas with 2500 mm of precipitation and with annual evapotranspiration of 1000 mm were considered humid, and upper mountain higher elevation areas were cold regions. Likewise, farmers from the Sonoran Desert identified zones in mesic environments for *Agave* cultivation at the Tucson and Phoenix basin (Homburg and Sandor, 2011; Fish and Fish, 2014). In the current climate, ancestral dryland farming knowledge and its legacies in the landscape offer alternatives for developing sustainable agricultural strategies to cultivate *Agave*, integrating suitable species while reducing the irrigation water required with rising temperatures and droughts (Ortiz-Cano *et al.*, 2020; Davis *et al.*, 2022).

Cultivation of *Agave* has evolved from a subsistence farming strategy to an industrial crop, highly specialized and with a sophisticated monoculture scheme (Borland *et al.*, 2011; Davis *et al.*, 2011; Garcia-Moya *et al.*, 2011; Cushman *et al.*, 2015). Drought tolerance, CAM metabolism and broad genetic diversity of *Agave* enable species to adapt to unpredictable rainfall conditions in the driest regions of North America and offer an alternative crop with potential to adapt to future warming and drought conditions (Davis *et al.*, 2022). However, accelerated agro-industrial development and inflated demand of *Agave* subproducts threaten *Agave* ecosystems and biodiversity (Stewart *et al.*, 2015). Regions such as the borderlands of Mexico and the south-western USA play an important role for the future of *Agave* as a crop (Borland *et al.*, 2011; Lewis *et al.*, 2015). The abundant legacies of ancestral traditional ecological knowledge and cultural connection with *Agave* as a crop open opportunities for direct application of regenerative agriculture strategies for cultivating *Agave* and for conservation of biodiversity in the region (Levin, 2022). For example, *Agave* silvopastoral systems, in combination with tissue culture to mass produce plants, have shown potential for conservation efforts of *A. angustifolia* in the current drought and climate in Sonora, Mexico (Cervantes Mendívil *et al.*, 2007; Sánchez *et al.*, 2020). Likewise, *Agave* agroforestry and traditional ecological knowledge could revitalize small rural economies and, alternatively, create sustainable management strategies to preserve fragile *Agave* ecosystems (Torres-García *et al.*, 2019).

## *AGAVE* DEVELOPMENT FOR THE FUTURE

When evaluating the potential for *Agave* production in the future, it might be useful to consider the historical development of a commercial cropping system with similar ancestral roots. Today, corn (*Zea mays*) is the most widely produced cereal crop in the world, but its agricultural origins date back to pre-Columbian times (García-Lara and Serna-Saldivar, 2019), similar to *Agave*. Corn and *Agave* originate from similar geographical regions, and there are 64 races of corn associated with Mexico (García-Lara and Serna-Saldivar, 2019). In the near future, the environmental change associated with climate might require diversification of crops and the adoption of more climate-resilient alternatives, such as *Agave*. Just as the traditional knowledge of corn guided agricultural development in North America over the last two centuries, there is a legacy of traditional knowledge around *Agave* that can provide a foundation for developing climate-resilient agriculture in the region.

*Agave* species might have more diversified uses than corn, but they can also be a source of animal feed, food, sweeteners and biofuel (the primary products made from corn). The cultural legacy of *Agave* provides traditional knowledge about benefits of these CAM plants that can support human health in a changing climate. Despite the tremendous historical success of corn as an agricultural product, yields are expected to decline in some regions as climate change continues (Bezner Kerr *et al.*, 2022). Cultural histories of the Americas identify corn as an important subsistence crop in the borderland region of Sonoran Arizona and Mexico (Katz *et al.*, 1974). *Agave* species were equally important in this region, although more concentrated in the southerly portion of the Sonoran Desert (Gentry, 1982). With atmospheric warming and increasing intensity of drought in this region, the northern range for some *Agave* species will likely expand (Davis *et al.*, 2021, 2022) and can, potentially, offset losses of other cropping systems in the northern Sonoran Desert region.

New work revitalizing traditional knowledge of regenerative agricultural practices indicates that *A. angustifolia* and similar relatives (*A. americana*, *A. lechuguilla*, etc.) can thrive in agroforestry systems or with specialized soil amendments. Intercropping *Agave* and mesquite trees, for example, allows trees to help maintain the soil moisture balance with deep-rooted systems that can draw water to the soil surface and canopy shading that prevents soil evaporative losses (Nabhan *et al.*, 2020; Fig. 4). This strategy provides resilient crop production in times of extreme drought, while also fostering a carbon mitigation strategy (Lehner and Rosenberg, 2017). Rock mulching can also be used to retain soil moisture, as described in the previous section, and is another traditional practice that was recently described in sites in the borderland region of the southwestern USA (Fish *et al.*, 1985; Ortiz-Cano *et al.*, 2020). Rock mulch reduces the evaporative losses from soil, making more water available for plant growth (e.g. Diaz *et al.*, 2005).

Traditional knowledge can inform both land management and nutritional opportunities associated with ancient crops. After centuries of agricultural development in the USA, it was discovered that ancient and traditional processing of corn with alkali results in increased nutritional value of corn-based products (Katz *et al.*, 1974). A review of traditional practices for managing and processing *Agave* reveals that there are health benefits of these plants that are under-realized (Davis *et al.*, 2019). *Agave* species have been identified as having anti-inflammatory and anti-carcinogenic properties (Colunga-GarcíaMarín and May-Pat, 1993; Yokosuka *et al.*, 2000; Jin *et al.*, 2004; Hackman *et al.*, 2006; Garcia-Morales *et al.* 2022). Traditional methods of roasting the plants in earthen pits might contribute to the beneficial health properties of fermented products. Even without fermentation, traditionally roasted leaves of *A. americana* have nutritional value owing to the greater protein and increased digestibility of other nutrients (Laferriere *et al.*, 1991) after roasting. *Agave* plants can also be a substantial source of calcium and iron (Laferriere *et al.*, 1991).

## CONCLUSION

Crassulacean acid metabolism is expressed prominently in the *Agave* genus, and the traits associated with this physiological condition allow for many products valued by people, e.g. sugar, medicine, fibre and food. The sugar, medicinal compounds and vitamins beneficial for humans are made possible by the storage of soluble carbohydrates and secondary compounds in the large vacuoles of these succulent plants. The large vacuoles allow for 24 h carbon cycling that is fundamental for CAM plants. Leaf structures associated with succulent CAM plants are adapted to maintain rigidity and turgor pressure in extreme drought and heat. The water-use efficiency associated with CAM is made possible by both water storage and hydraulic isolation of the plants, requiring strong but flexible fibres to support the mass of water inside the plant, while preventing contact between the plant tissue and the soil. The long cellulosic fibres that support this leaf structure are desirable for weaving textiles and ropes.

The concentrated sugars in the plant tissues of *Agave* plants are also valuable for fermented beverages and spirits. Although the profitability of *Agave* spirits has led to investment in agricultural production of *A. tequilana* and *A. angustifolia* that is internationally acknowledged (tequila and bacanora), other potentially valuable products generated from the many known species of *Agave* are less recognized. The Appellation of Origin now protects six different mescals from exploitation in non-native regions, but further agricultural development of *Agave* in the borderlands for food, sugar, fibre and medicine has promise. The political, ecological and agronomic history of *Agave* in the region suggests that there is great potential for this group of CAM plants to provide climate-resilient agricultural resources. The water-use efficiency, drought tolerance and heat tolerance made possible by CAM physiology have allowed *Agave* species to provision humans for thousands of years, and these traits continue to hold promise for a future with changing climate.
